# Application of protective weight-bearing in osteonecrosis of the femoral head: A systematic review and meta-analysis of randomized controlled trials and observational studies

**DOI:** 10.3389/fsurg.2022.1000073

**Published:** 2022-11-02

**Authors:** Wen-huan Chen, Wen-xuan Guo, Jian-xiong Li, Qiu-shi Wei, Zi-qi Li, Wei He

**Affiliations:** ^1^ The Third Clinical Medical School, Guangzhou University of Chinese Medicine, Guangzhou, China; ^2^ The First Clinical College, Zhejiang Chinese Medical University, Hangzhou, China; ^3^ The First Clinical Medical School, Guangzhou University of Chinese Medicine, Guangzhou, China; ^4^ Department of Joint Surgery, The Third Affiliated Hospital of Guangzhou University of Chinese Medicine, Guangzhou, China

**Keywords:** osteonecrosis of the femoral head, protective weight-bearing, conservative treatment, hip preservation treatment, nonsurgical, prognosis

## Abstract

**Background:**

The aim of this systematic review and meta-analysis was to estimate the efficacy and prognostic value of protective weight-bearing for ONFH.

**Methods:**

The authors searched the PubMed, EMBASE and Cochrane Library databases, up to February 25, 2022. RCTs and observational studies on conservative treatment, including the use of crutches, for skeletally mature patients with ONFH and written in English were included. Outcomes were the total hip arthroplasty (THA) rate, collapse rate, Hip Harris score (HHS) and visual analog scale (VAS) score. Cochrane Review Manager Software 5.4 and Stata 15.1 were used to perform the statistical analyses.

**Results:**

A total of 14 studies involving 813 patients (1,025 hips) were included in this meta-analysis. The results showed that the THA rate, collapse rate, HHS and VAS scores in the protective weight-bearing group were not significantly different from those in the surgical group. In the protective weight-bearing group, the results showed that the THA rate was 40%, 8% in ARCO stage II, 37% in ARCO stage III, and the collapse rate was 46%. The mean HHS and VAS score was 80.86 and 1.00, respectively. The HHS score at the 3-, 6-, 12-, and 24-month follow-up was 79.93, 83.94, 85.94, and 96.09 points, respectively, whereas the VAS score at the 6- and 12-month follow-up was 2.20 and 1.29, respectively.

**Conclusion:**

Protective weight bearing could achieve satisfactory results in terms of THA rate, collapse rate, HHS and VAS scores. Protective weight-bearing allows most precollapse patients to preserve the hip but also allows postcollapse patients to delay THA or hip-preserving surgery. The effects and prognosis of protective weight-bearing in the short or mid-term are noninferior to surgical hip preservation and are a viable alternative option for osteonecrosis of the femoral head.

## Introduction

Osteonecrosis of the femoral head (ONFH) is a destructive disease of the joint that often severely affects the patient's function and reduces the quality of life (1). In China, an estimated 8.12 million patients are older than age 15 (2). The incidence of ONFH increases each year, thus suggesting its emergence as a problem that cannot be underestimated in society or the world.

Although total hip arthroplasty (THA) is considered the ultimate treatment option for advanced osteoarthritis (OA) secondary to femoral head collapse (3), the reported rate of THA decreased from 2009 to 2015 (93.56%–89.52%) due to various potential complications (4). For younger patients, joint-preserving therapies seem to be a better approach than THA because younger and more active adults may require multiple revision surgeries (5, 6). However, the results of hip preservation surgery are always controversial (7–9). Moreover, multiple surgeries place a considerable financial burden on the patient, their family and society (8, 10, 11). Therefore, conservative treatment might be the best first step in the stepwise treatment of ONFH. In fact, 50% of patients in mainland China would prefer to undergo nonsurgical treatment (1).

Conservative treatment includes a combination of interventions, including shockwave therapy, medication, protective weight-bearing, and physical therapy (12, 13). In previous studies, researchers reported the general futility of conservative treatment in delaying progression to THA (14) and indicated that protective weight-bearing can only alleviate symptoms, such as pain (13, 15, 16). In several recent studies, researchers have shown that the clinical efficacy of conservative treatment is not inferior to that of hip preservation surgery (17–19). Protective weight-bearing is the most straightforward method used to relieve stress on the femoral head, and it is readily accepted by doctors and patients and recommended in many guidelines (13, 20, 21). However, this approach has not received sufficient attention, and evidence for its effectiveness is lacking.

Therefore, the aim of this meta-analysis is to evaluate the efficacy and prognostic value of protective weight-bearing for patients with ONFH as an option for clinical treatment.

## Methods

### Search strategy and selection criteria

This study was performed according to the Preferred Reporting Items for Systematic Reviews and Meta-analyses (PRISMA) guidelines (22). We registered our review in the PROSPERO database (registration number: CRD42022313170). Randomized controlled trials (RCTs) and observational studies reporting on protective weight-bearing in ONFH were included. Two reviewers independently conducted a search in the PubMed, EMBASE and Cochrane Library databases using the same strategy. The last search was conducted on February 25, 2022 (detailed search strategies are reported in Table 1 and Supplementary Material S2). When screening the literature, researchers read the full text to determine whether protective weight-bearing was used. Because protective weight bearing is a conservative treatment method, the key words included “osteonecrosis of the femoral head”, “femur head necrosis”, “conservative treatment”, “nonoperative” and “nonsurgical” to perform a more comprehensive search of the literature.

**Table 1:** Search details.

Databases	Strategy	*n*
PubMed	(“femur head necrosis”[MeSH Terms] OR (“femur”[All Fields] AND “head”[All Fields] AND “necrosis”[All Fields]) OR “femur head necrosis”[All Fields] OR (“legg calve perthes disease”[MeSH Terms] OR (“legg calve perthes”[All Fields] AND “disease”[All Fields]) OR “legg calve perthes disease”[All Fields] OR (“osteonecrosis”[All Fields] AND “femoral”[All Fields] AND “head”[All Fields]) OR “osteonecrosis of the femoral head”[All Fields])) AND (“nonop”[All Fields] OR “nonoperative”[All Fields] OR “nonoperatively”[All Fields] OR (“nonsurgical”[All Fields] OR “nonsurgically”[All Fields]) OR (“conservative treatment”[MeSH Terms] OR (“conservative”[All Fields] AND “treatment”[All Fields]) OR “conservative treatment”[All Fields]))	517
Embase	(“femur head necrosis”/exp OR “femur head necrosis” OR ((“femur”/exp OR femur) AND (“head”/exp OR head) AND (“necrosis”/exp OR necrosis))) AND (“conservative treatment”/exp OR “conservative treatment”)	662
Cochrane Library	(osteonecrosis of the femoral head OR Femur head necrosis) AND (conservative treatment OR (nonoperative OR nonsurgical))	15

Both comparative and noncomparative studies written in English that reported conservative treatment for skeletally mature patients with ONFH were included. Conference abstracts, case reports, editorial comments, animal studies, letters, duplicate publications, studies with patients younger than 18 years old, studies lacking full-text or outcome data, and studies in which protective weight-bearing was not mentioned in specific measures were excluded. Two independent researchers screened the study titles and abstracts. The full text of the studies potentially meeting the eligibility criteria was retrieved for a more detailed read to make a final decision.

### Quality assessment

Two reviewers independently assessed the methodological quality of all included studies using the Methodological Index for Non-Randomized Studies (MINORS). In total, 12 items were evaluated, and each item was divided into 0–2 points. The first to eighth items were for noncomparative studies, and the last 4 items were for comparative studies. The risk-of-bias tool of the Cochrane Collaboration was used to assess the methodologic quality of RCTs. The 7 items used to evaluate bias in each trial included randomization sequence generation, allocation concealment, the blinding of participants and personnel, the blinding of outcome assessments, incomplete outcome data, selective reporting, and other biases, such as the baseline characteristics between different groups.

### Data extraction

A researcher extracted the following data for the study: (1) basic information of the included studies: the first author, publication year, country, study design type, staging and classification of ONFH, study group, intervention, sex and age, number of patients and hips, and follow-up time; (2) the key elements of risk of bias assessment; and (3) the primary outcomes (THA rate and the collapse rate of the femoral head) and secondary outcomes (Hip Harris score and visual analog scale).

At present, a unified standard for the conservative treatment of ONFH is lacking. Although many guidelines and consensuses clearly state that limiting the weight-bearing of the affected limb helps treatment, we have not identified a clear definition of protective weight-bearing. Therefore, it was defined in this study as the clearly indicated use of walking aids, such as crutches and canes, in the conservative treatment group or a clear stipulation that patients should reduce weight-bearing of the affected limb when walking for a period of time.

### Outcome measures

The primary outcomes were the rate of THA and the rate of femoral head collapse progressing >2 mm at final follow-up. The secondary outcomes were the Hip Harris score (HHS) and visual analog scale (VAS).

### Statistical analysis

The Stata statistical software (Stata version 15.1, StataCorp, TX, USA) and Review Manager software (RevMan version 5.4, the Nordic Cochrane Centre, the Cochrane Collaboration, Copenhagen, Denmark) were used for data management and statistical analysis. If the mean, percentage or SD were not described in the original study, they were calculated from the original data given in the text or provided in the appendix.

The relative risk (RR) was used as the effect analysis statistic for dichotomous variables, and the weighted mean difference (WMD) was used as the effect analysis statistic for continuity variables. Each effect quantity is reported as a 95% CI. The Mantel‒Haenszel method was used for dichotomous variables, and the inverse variance method was used for continuous variables. In the overall effect *Z*-test, *P* < 0.05 was considered statistically significant. The heterogeneity between the included research results was analyzed with the *x*^2^ test (the test level was set as *α *= 0.1) and combined with *I*^2^ to quantitatively judge the size of heterogeneity. If statistical heterogeneity was absent among the research results, the fixed effect model was used to conduct a meta-analysis; if statistical heterogeneity was observed among the research results, the random effect model was used to conduct a meta-analysis. Significant clinical heterogeneity was addressed with a subgroup analysis, sensitivity analysis, or only a descriptive analysis. The inspection level of the meta-analysis was set to *α *= 0.05.

## Results

### Search

**Figure 1:**
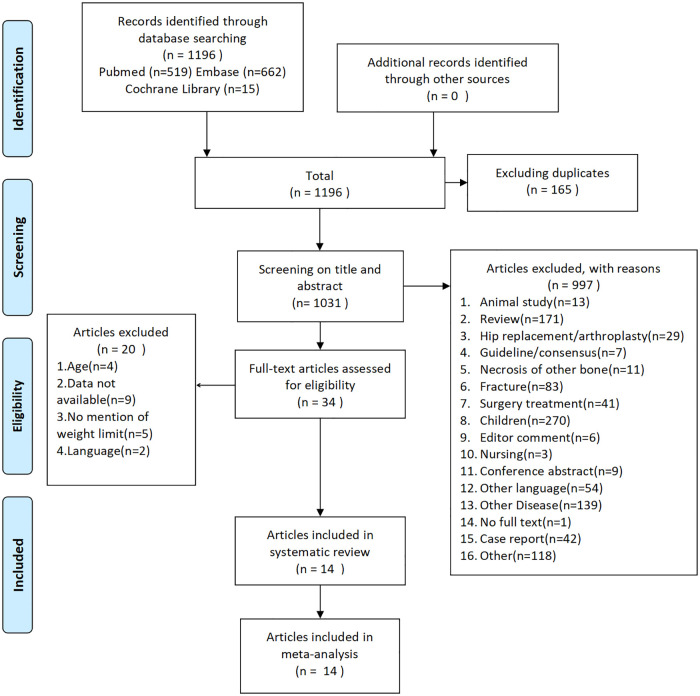
Flow diagram showing the study selection process.

When the number of patients or affected hips was not clearly reported in the text and only one of these parameters was provided, we considered all patients included in the literature to have only disease in one hip during the statistics. Based on a search of published studies, 1,196 potentially eligible records were identified, and the full texts of 34 studies were reviewed. Of these studies, 14 trials met the inclusion criteria and were retained, whereas the remaining studies were excluded for various reasons (Figure 1).

### Baseline characteristics

Of the 14 included studies, 3 were RCTs (8, 23, 24), 6 were noncomparative studies (25–30) and 5 were comparative studies (18, 31–34). A total of 813 patients (1,025 hips) with a mean age of 38.1 years were reported, and most patients were male (59.19%). These studies explicitly described the use of crutches for protective or limited weight-bearing in all conservative treatment groups. The baseline characteristics of the included studies are presented in Table 2.

**Table 2:** Baseline characteristics of the included studies.

First author	Year	Country	Study design	Stage of ONFH	Groups	Inventions	Patients (Hips)	Sex (M: F)	Age (mean ± SD, years)	Follow-up time (mean, months)
Algarni (25)	2018	Arabia	Non-comparative study	ARCO I, II	Conservation	Crutches	21 (33)	9:12	37.5	60
Churchill (26)	1991	UK	Non-comparative study	Ficat III	Conservation	Crutches	29 (35)	10:19	37.5	60
Fang (31)	2020	China	Comparative study	Ficat I, II	Conservation	Crutches	30 (41)	26:4	48.1	33.5
					Surgery	CD+ tantalum	30 (41)	26:4	44.2	
Huang (27)	2020	China	Non-comparative study	ARCO I–IV	Conservation	Crutches	33 (66)	9:24	42.5	178.56
Koo (8)	1995	South Korea	RCT	Steinberg I–III	Conservation	Crutches	19 (19)	31:2	47	24–45
					Surgery	CD	18 (18)			
Steinberg (32)	1990	USA	Comparative study	Steinberg I–III	Conservation	Crutches	55 (55)	No	No	24–48
					Surgery	CD	40 (40)	30:10	No	
Sun (33)	2014	China	Comparative study	ARCO I–III	Conservation	Crutches	87 (127)	26:61	33	74.4
					Surgery	Bone graft	42 (72)	19:23	30.7	85.2
Tomaru (18)	2021	Japan	Comparative study	JIC A, B, C1, C2	conservation	Crutches	33 (33)	2:31	35.7	104.4
					Surgery	CD	33 (33)	2:31	35.1	70.8
Vulpiani (28)	2012	Italy	Non-comparative study	ARCO I–III	Conservation	Crutches	36 (36)	23:13	48.9	24
Wiesmann (29)	1998	Germany	Non-comparative study	No	Conservation	Crutches	17 (24)	9:8	29.41	36.47
Wu (30)	2018	China	Non-comparative study	ARCO II	Conservation	Crutches	168 (202)	141:27	47.07	91
Stulberg (23)	1991	USA	RCT	Ficat	Conservation	Crutches	17 (26)	No	38.6	≥18
					Surgery	CD	19 (29)			
Wang (34)	2005	China	Comparative study	ARCO I–III	Conservation	Crutches	23 (29)	20:3	39.8	25.2
					Surgery	CD+ bone graft	25 (28)	23:2	39.9	25.8
Neumayr (24)	2006	USA	RCT	Steinberg	Conservation	Crutches	21 (21)	11:10	26.41	80
					Surgery	CD	17 (17)	8:9	24.67	

### Quality assessment

**Figure 2:**
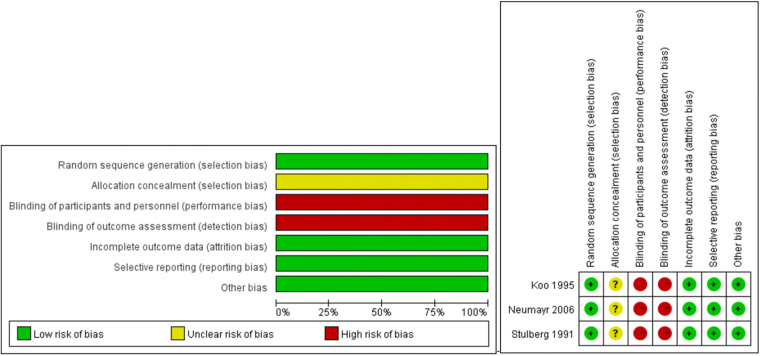
Methodological quality of the RCTs.

Only 3 studies were described as RCTs. The methodological quality of the RCTs according to the Cochrane Collaboration risk-of-bias criteria is shown in Figure 2. No trials reported the methods for allocation concealment. Blindness was difficult to achieve for the participants and personnel because of the nature of the treatment. The study protocols were not found, thus causing difficulty in assessing the reporting bias. Level 1b evidence was observed for the included RCTs based on the Oxford Centre for Evidence-based Medicine Levels of Evidence (Figure 2).

The mean MINORS score of all non-RCT studies was 13.45 ± 3.40. The comparative studies had a mean score of 17.2 ± 1.44, whereas this value was 10.33 ± 0.56 for noncomparative studies (Table 3).

**Table 3:** Results of the MINORS evaluation.

MINORS item	Algarni 2018	Churchill 1991	Fang 202	Huang 2020	Steinberg 1990	Sun 2014	Tomaru 2021	Vulpiani 2012	Wiesmann 1998	Wu 2018	Wang 2005
1. A clearly stated aim	2	2	2	2	2	2	2	2	2	2	2
2. Inclusion of consecutive patients	2	2	2	2	1	2	2	2	2	2	2
3. Prospective collection of data	0	0	0	0	0	2	0	0	0	2	0
4. Endpoints appropriate to the aim of the study	2	2	2	2	2	2	2	2	2	2	2
5. Unbiased assessment of the study endpoint	0	0	0	0	0	0	0	0	0	0	0
6. Follow-up period appropriate to the aim of the study	2	2	1	2	2	2	2	2	2	2	2
7. Loss to follow up less than 5%	2	2	2	2	2	2	2	2	2	2	2
8. Prospective calculation of the study size	0	0	0	0	0	0	0	0	0	0	0
9. An adequate control group	-	-	2	-	2	2	2	-	-	-	2
10. Contemporary groups	-	-	2	-	0	2	1	-	-	-	2
11. Baseline equivalence of groups	-	-	2	-	1	2	2	-	-	-	2
12. Adequate statistical analyses	-	-	2	-	2	2	2	-	-	-	2
Total score	10	10	17	10	14	20	17	10	10	12	18

### THA rate

**Figure 3:**
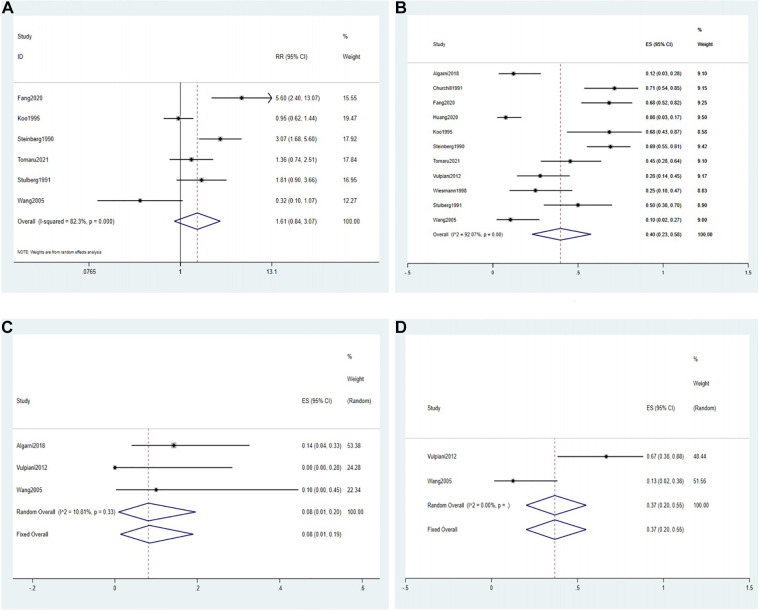
Forest plot of the THA rate. (**A**) The forest plot of the random effects model for 2 RCTs and 4 comparative studies; (**B**) the forest plot of the random effects model for a single-arm meta-analysis of 11 studies; (**C**) the forest plot of the fixed-effects model for a single-arm meta-analysis of 3 studies (ARCO stage II); (**D**) the forest plot of the fixed-effects model for a single-arm meta-analysis of 2 studies (ARCO stage III).

The THA rate was reported in 2 RCTs, 4 comparative studies and 5 noncomparative studies (8, 18, 23, 25–29, 31, 32, 34). Based on all RCTs and comparative studies, a meta-analysis with the random effect model (Figure 3A) showed that the THA rate in the conservative group was not significantly different from that in the surgical group (RR = 1.61, 95% CI: 0.84–3.07, *P *> 0.05), and these data exhibited high heterogeneity (*I*^2^ = 82.3%).

A single-arm meta-analysis of 11 studies reporting THA rates in the conservative group was performed based on a random effects model, resulting in a pooled effect size (ES = 0.40, 95% CI: 0.23–0.58, *I*^2^ = 92.07%) (Figure 3B).

The THA rate of patients with Association Research Circulation Osseous (ARCO) stage II in the conservative group was reported in 3 studies. Algarni et al. (25) reported that the THA rate was 14%. Vulpiani et al. (28) reported that it was 0, whereas Wang et al. (34) reported it was 10%. A single-arm meta-analysis with the fixed effect model showed that the THA rate in ARCO stage II was 8% (ES = 0.08, 95% CI: 0.01–0.20, *I*^2^ = 10.81%) (Figure 3C).

Moreover, 2 studies reported the THA rate of patients with ARCO stage III in the conservative group. Vulpiani et al. (28) reported that this rate was 67%, whereas Wang et al. (34) reported it was 12.5%. A single-arm meta-analysis with the fixed effect model showed that the THA rate in ARCO stage III was 37% (ES = 0.37, 95% CI: 0.20–0.55, *I*^2^ = 0%) (Figure 3D).

### Collapse rate

**Figure 4:**
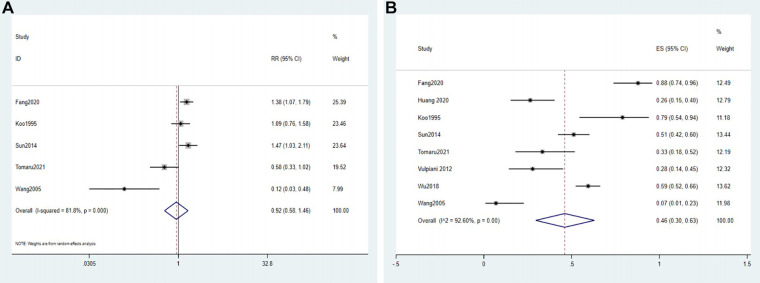
Forest plot of the collapse rate. (**A**) The forest plot of the random effects model for 1 RCT and 4 comparative studies; (**B**) the forest plot of the random effects model for a single-arm meta-analysis of 8 studies.

The collapse rate was reported in 8 included studies (8, 18, 27, 28, 30, 31, 33, 34). Based on 1 RCT and 4 comparative studies, we compared the collapse rates in the conservative and surgical groups. Because of the high heterogeneity (*I*^2^ = 81.8%), we selected the random effects model. The results showed that the conservative group and the surgical group did not significantly differ (RR = 0.92, 95% CI: 0.58–1.46, *P* > 0.05) (Figure 4A).

Based on 8 studies, the meta-analysis with the random effects model in the conservative group showed that the total effect size was 0.46 (95% CI: 0.30–0.63, *I*^2^ = 92.6%) (Figure 4B).

### Hip Harris score

**Figure 5:**
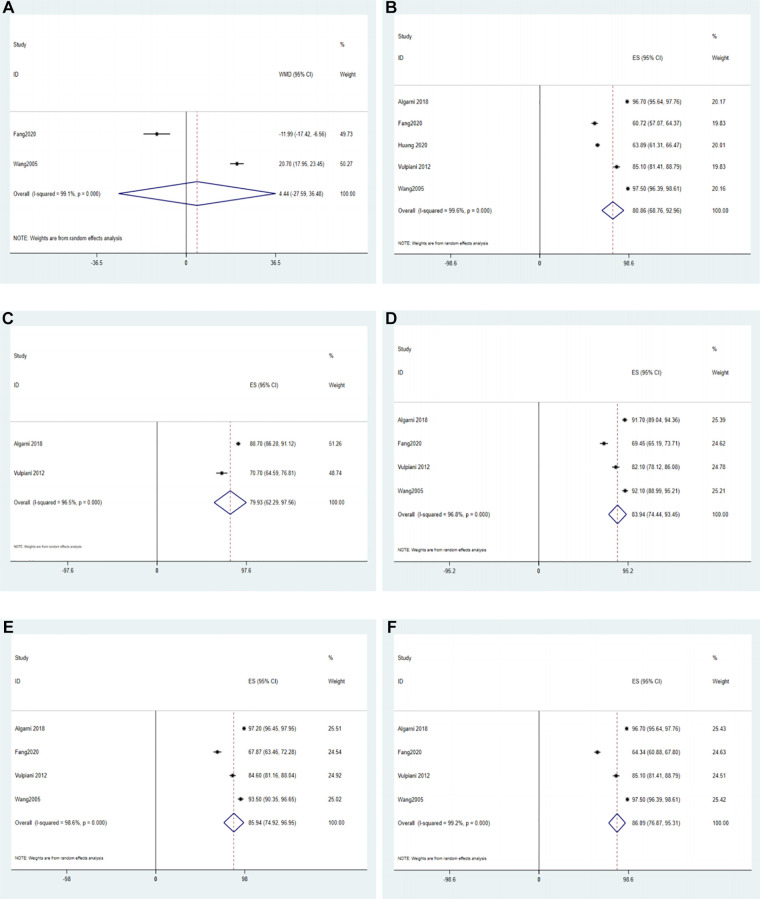
The forest plot of HHS. (**A**) The forest plot of the random effects model for 2 comparative studies; (**B**) the forest plot of the random effects model for a single-arm meta-analysis of 5 studies; (**C**) the forest plot of the random effects model for a single-arm meta-analysis of 2 studies at 3-month follow-up; (**D**) the forest plot of the random effects model for a single-arm meta-analysis of 4 studies at 6-month follow-up; (**E**) the forest plot of the random effects model for a single-arm meta-analysis of 4 studies at 12-month follow-up; (**F**) the forest plot of the random effects model for a single-arm meta-analysis of 4 studies at 24-month follow-up.

Five studies reported on the HHS (25, 27, 28, 31, 34). A meta-analysis with the random effects model of 2 comparative studies did not show a significant difference between the conservative group and the surgical group in the HHS (WMD = 4.44, 95% CI: −27.95–36.48), and these data exhibited high heterogeneity (*I*^2^ = 99.1%) (Figure 5A). One study (24) reported the results of the Children's Hospital Oakland Hip Evaluation Scale (CHOHES), which is a modification of the HHS, in the surgical group and conservative group. The mean clinical improvement was 18.1 points for the surgical group compared with 15.7 points for the conservative group, and this difference was not significant.

Based on the 5 studies that reported on the HHS in the conservative group, a single-arm meta-analysis with a random effects model showed that the ES was 80.86 (95% CI: 68.76–92.96, *I*^2^ = 99.6%) (Figure 5B).

The results of HHS were reported in two studies after 6, 12, and 24 months of follow-up in the conservative and surgical groups (31, 34). A random effects model did not show statistically significant differences in the HHS between patients in the conservative and surgical groups at different follow-up times (*P *> 0.05) (Supplementary Material S1).

Moreover, 2 studies reported the results of the HHS at the 3-month follow-up in the conservative group. Algarni et al. (25) reported an HHS of 88.7 ± 7.1, whereas Vulpiani et al. (28) reported an HHS of 70.7 ± 17.9. A single-arm meta-analysis with the random effect model showed that the HHS at the 3-month follow-up was 79.93 (ES = 79.93, 95% CI: 62.29–97.56, *I*^2^ = 96.5%) (Figure 5C).

The results of HHS at the 6-month follow-up in the conservative group were reported in 4 studies. Specifically, Algarni et al. (25), Fang et al. (31), Vulpiani et al. (28) and Wang et al. (34) reported HHS values of 91.7 ± 7.8, 69.45 ± 13.92, 82.1 ± 11.5, and 92.1 ± 8.4, respectively. Thus, a single-arm meta-analysis with the random effect model showed that the HHS at the 6-month follow-up was 83.94 (ES = 83.94, 95% CI: 74.44–93.45, *I*^2^ = 96.8%) (Figure 5D).

Four studies reported the results of HHS at the 12-month follow-up in the conservative group. Specifically, Algarni et al. (25), Fang et al. (31), Vulpiani et al. (28) and Wang et al. (34) reported HHS values of 97.2 ± 2.2, 67.87 ± 14.42, 84.6 ± 9.3, and 93.5 ± 8.5, respectively. Thus, a single-arm meta-analysis with the random effect model showed that the HHS at the 12-month follow-up was 85.94 (ES = 85.94, 95% CI: 74.92–96.95, *I*^2^ = 98.6%) (Figure 5E).

The results of HHS at the 24-month follow-up in the conservative group were reported in 4 studies. Specifically, Algarni et al. (25), Fang et al. (31), Vulpiani et al. (28) and Wang et al. (34) reported HHS values of 96.7 ± 3.1, 64.34 ± 11.29, 85.1 ± 9.6, and 97.5 ± 2.9, respectively. Thus, a single-arm meta-analysis with the random effect model showed that the HHS at the 24-month follow-up was 86.09 (ES = 86.09, 95% CI: 76.87–95.31, *I*^2^ = 99.2%) (Figure 5F).

### VAS score

**Figure 6:**
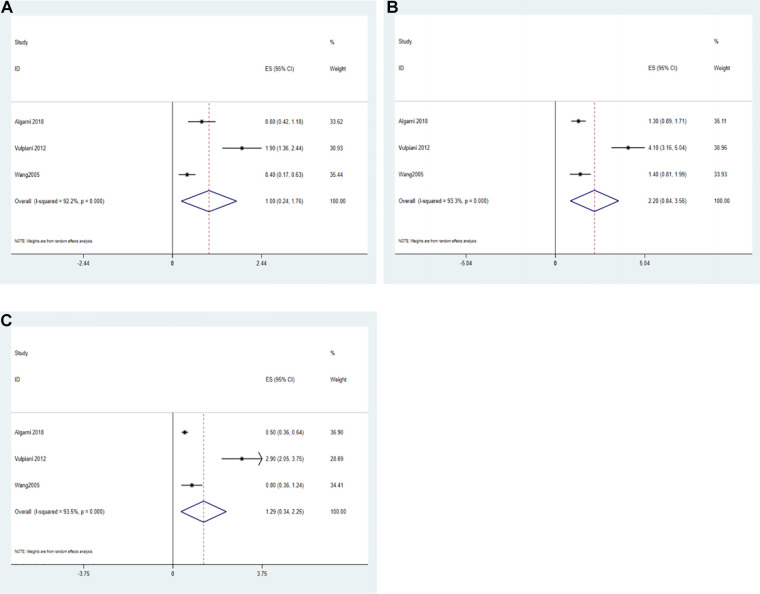
Forest plot of VAS. (**A**) The forest plot of the random effects model for a single-arm meta-analysis of 2 studies; (**B**) the forest plot of the random effects model for a single-arm meta-analysis of 3 studies at 6-month follow-up; (**C**) the forest plot of the random effects model for a single-arm meta-analysis of 3 studies at 12-month follow-up.

Only three included studies reported VAS results (25, 28, 34). Wang et al. (34) reported that the mean VAS scores were 0.4 and 4.7 in the conservative group and surgical group, respectively. Taken together with 2 noncomparative studies, a single-arm meta-analysis with a random effects model showed that the VAS score in the conservative group was 1.00 (95% CI: 0.24–1.76, *I*^2^ = 92.2%) (Figure 6A).

The VAS results at the 6-month follow-up in the conservative group were reported in 3 studies. Algarni et al. (25), Vulpiani et al. (28) and Wang et al. (34) reported values of 1.3 ± 1.2, 4.1 ± 2.7, and 1.4 ± 1.6, respectively. Thus, a single-arm meta-analysis with the random effect model showed that the VAS score at the 6-month follow-up was 2.20 (ES = 2.20, 95% CI: 0.84–3.56, *I*^2^ = 93.3%) (Figure 6B).

The VAS results at the 12-month follow-up in the conservative group were reported in 3 studies. Algarni et al. (25), Vulpiani et al. (28) and Wang et al. (34) reported values of 0.5 ± 0.4, 2.9 ± 2.3, and 0.8 ± 1.2, respectively. Thus, a single-arm meta-analysis with the random effect model showed that the VAS score at the 12-month follow-up was 1.29 (ES = 2.29, 95% CI: 0.34–2.25, *I*^2^ = 93.5%) (Figure 6C).

## Discussion

Whether the efficacy and prognostic value of hip preservation surgery are superior to those of protective weight-bearing treatment for patients pre- or postcollapse is difficult to determine due to a lack of high-quality studies that specifically evaluate the outcome. In this meta-analysis, the THA rates, collapse rates, and clinical function scores did not significantly differ between the protective weight-bearing group and hip preservation surgery group. In addition, this meta-analysis showed that patients with ARCO stage II had a lower rate of THA after protective weight-bearing treatment than patients with ARCO stage III.

The rate of THA is one of the most important outcome indicators in the study of hip preservation methods. Core decompression (CD) is the most common surgery for the treatment of ONFH prior to collapse, and the THA rate at 26 months after surgery is approximately 38% (35–37). Previous studies have shown that the THA rates do not significantly differ between nonsurgical and CD treatments (17). This finding was also observed in this study, and the THA rate with protective weight-bearing was approximately 40%. Although the THA rate was much higher in patients after collapse (ARCO Stage III, 37%) than in patients before collapse (ARCO Stage II, 8%), the results still increased the confidence of patients—even if they had experienced collapse, nonsurgical treatment could still delay arthroplasty or hip preservation surgery. The collapse rate of the femoral head reflects the progression of ONFH, and severe collapse often leads to OA. Some patients choose THA, while some patients who experience collapse can maintain good clinical function without surgery; that is, this strategy can achieve a survival situation for patients who experience collapse (30, 38). This effect may be attributable to the following: after the stress of the femoral head is reduced, revascularization is accelerated in the femoral head, and the use of anti-osteoporotic drugs can reduce bone absorption and promote bone restoration in the femoral head (39, 40). For postcollapse patients, bone restoration in the femoral head cannot completely restore the sphericity of the femoral head but can only increase the density and diameter of bone trabeculae to stop further collapse (41, 42). During the repair process, the acetabulum and femoral head eventually form an adaptive state, which is not greatly affected by the presence of arthritis (43, 44).

In addition to the THA rate and collapse rate, clinical improvement in terms of function and pain was evaluated in this study using the HHS and VAS. In a previous meta-analysis, Mont defined an HHS > 80 as clinical success and reported a higher clinical success rate in the surgical hip preservation group (64%) than in the nonsurgical treatment group (23%) (15). However, in a recent network meta-analysis, the HHS of the hip preservation surgery group was not significantly higher than that of the nonsurgical treatment group (17). Neumayr et al. (24) used the modified HHS score and found that improvements in the scores did not significantly differ between the two groups. The results of this study also suggest that the HHS outcome of the protective weight-bearing group was not significantly lower than that of the surgical group in the early and mid-term after treatment. The mean HHS result of protective weight-bearing was 80.86, and the VAS was 1.00. After a single-arm meta-analysis, the HHS results of protective weight-bearing at 3, 6, 12, and 24 months of follow-up were 79.93, 83.94, 85.94, and 96.09 points, respectively, whereas the VAS results at 6 and 12 months of follow-up were 2.20 and 1.29, respectively. Based on the functional outcomes at follow-up, protective weight-bearing may be a beneficial conservative treatment. Because Professor Mont's meta-analysis was published too early and the HHS results were presented as a success rate, obtaining the specific scores of the original studies was difficult. Therefore, we did not include these studies. Some studies of conservative treatment did not specify whether protective weight-bearing, such as using crutches, was performed, and we did not include these studies either. Weight-bearing will not increase the incidence of nontraumatic ONFH, but it may aggravate the collapse of the femoral head caused by necrosis (45). The concentration of stress in the hip joint after weight-bearing is one of the main mechanisms of collapse. A finite element study showed that when the stress on the femoral head exceeds 2.7801 MPa, the femoral head is prone to collapse (46). Uncontrolled collapse can lead to severe OA, pain, and functional limitation, and avoiding or controlling the collapse of the femoral head to an acceptable level is the core goal of all hip preservation treatments. In 2021, Yu et al. reported a higher hospitalization cost for patients with ONFH undergoing surgical treatment, including hip preservation surgery and THA (47). According to the results of this study, we propose protective weight-bearing as a cost-effective treatment to directly reduce stress in the hip joint.

This study first focused on one of the controversial points in nonsurgical treatment and reviewed nearly 30 years of published hip-preservation studies on protective weight-bearing. In addition, we included a large proportion of patients and assessed the results of early prognosis. The clinical results of different stages and follow-up times are listed in detail, which could help clinicians and patients gain a better overall understanding and judgment of the effectiveness and prognosis of protective weight-bearing.

This study has some limitations. First, most results were heterogeneous, which we attribute to the larger time span of the included studies. The dates of the included studies ranged from 1990 to 2021 and covered multiple countries and regions. This heterogeneity was difficult to address with a sensitivity analysis; in fact, we attempted this approach, but the results did not significantly change. Second, the specific mechanism of protective weight-bearing varied greatly. The differences were mainly in the timing of crutch use, which may also be a source of heterogeneity. This variability also illustrated the necessity for this study, which may contribute to the development of standardized protective weight-bearing strategies. Third, some studies were poor quality, such as no blinding. Last, there were many confounding factors in the treatment of nonsurgical hip preservation; for example, protective weight-bearing was often combined with other methods, follow-up time varied, and patients had different stages of ONFH. These may have led to a relatively lenient conclusion in our research.

## Conclusion

Low-certainty evidence suggests that protective weight-bearing is a viable alternative to hip-preserving surgery for ONFH. In this meta-analysis, protective weight-bearing achieved the same effect as surgical hip preservation in terms of THA rate, collapse rate, HHS and VAS. Protective weight-bearing allows most precollapse patients to preserve the hip but also allows postcollapse patients to delay THA or hip-preserving surgery. Moreover, we need more high-quality studies to further determine the efficacy of protective weight-bearing.

## Data Availability

The original contributions presented in the study are included in the article/**Supplementary Material**, further inquiries can be directed to the corresponding author/s.
