# Supplementary material for: Application of protective weight-bearing in osteonecrosis of the femoral head: A systematic review and meta-analysis of randomized controlled trials and observational studies
Source: Front Surg. 2022 Nov 2;9:1000073. doi: 10.3389/fsurg.2022.1000073 (PMC9667948; doi:10.3389/fsurg.2022.1000073)

**Supplementary materials 2**

Search Details

*Search string PubMed (n = 517)*

("femur head necrosis"[MeSH Terms] OR ("femur"[All Fields] AND "head"[All Fields] AND "necrosis"[All Fields]) OR "femur head necrosis"[All Fields] OR ("legg calve perthes disease"[MeSH Terms] OR ("legg calve perthes"[All Fields] AND "disease"[All Fields]) OR "legg calve perthes disease"[All Fields] OR ("osteonecrosis"[All Fields] AND "femoral"[All Fields] AND "head"[All Fields]) OR "osteonecrosis of the femoral head"[All Fields])) AND ("nonop"[All Fields] OR "nonoperative"[All Fields] OR "nonoperatively"[All Fields] OR ("nonsurgical"[All Fields] OR "nonsurgically"[All Fields]) OR ("conservative treatment"[MeSH Terms] OR ("conservative"[All Fields] AND "treatment"[All Fields]) OR "conservative treatment"[All Fields]))

*Search string Embase (n = 662)*

('femur head necrosis'/exp OR 'femur head necrosis' OR (('femur'/exp OR femur) AND ('head'/exp OR head) AND ('necrosis'/exp OR necrosis))) AND ('conservative treatment'/exp OR 'conservative treatment')

*Search string the Cochrane Library (n = 15)*

(osteonecrosis of the femoral head OR Femur head necrosis) AND (conservative treatment OR (nonoperative OR nonsurgical))

According to the search strategy, there were 37 articles, but in the Trials, only 15 articles met the requirements.


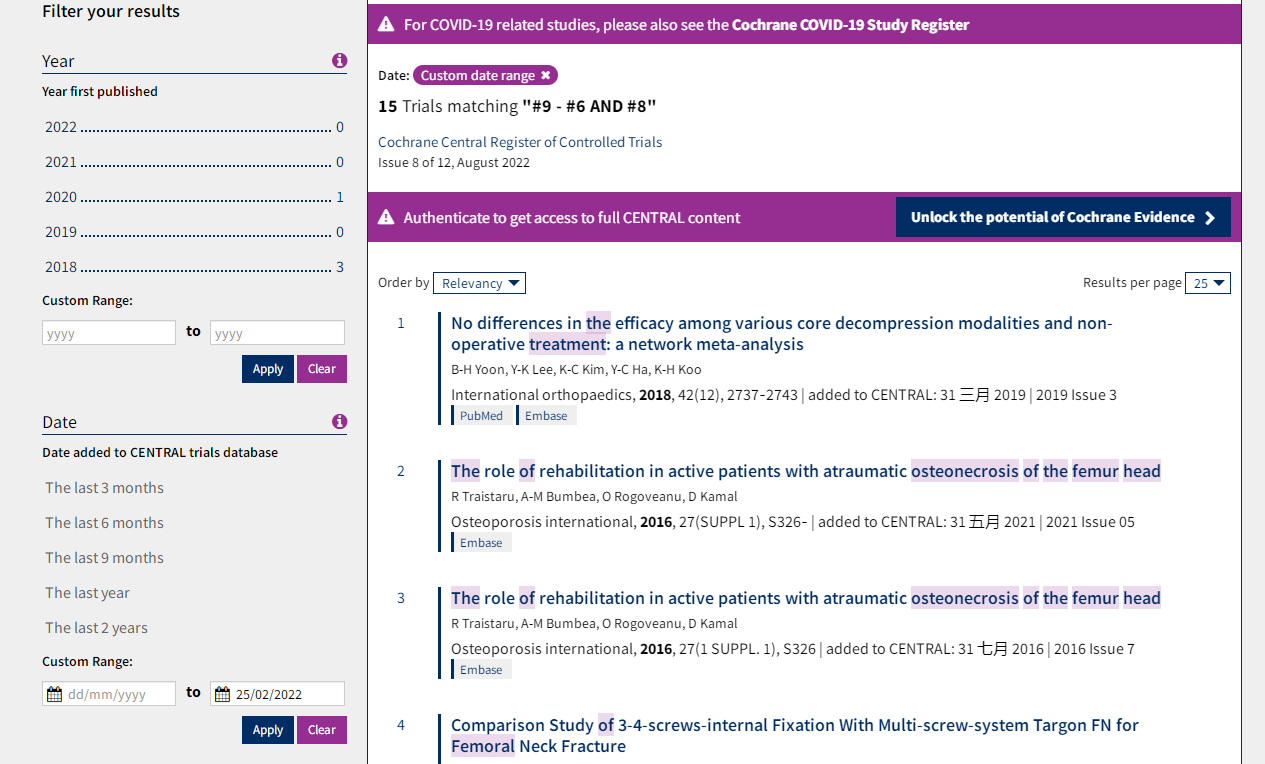

Supplement: Supplementary file 1 [file Datasheet1.docx]
